# RNA-Seq Analysis of Peripheral Whole Blood from Dairy Bulls with High and Low Antibody-Mediated Immune Responses—A Preliminary Study

**DOI:** 10.3390/ani13132208

**Published:** 2023-07-05

**Authors:** Xiuxin Zhao, Hanpeng Luo, Haibo Lu, Longgang Ma, Yanqin Li, Jinhuan Dou, Junxing Zhang, Yun Ma, Jianbin Li, Yachun Wang

**Affiliations:** 1 Ningxia Key Laboratory of Ruminant Molecular and Cellular Breeding, College of Animal Science and Technology, Ningxia University, Yinchuan 750021, China; zhaoxiuxin2003@163.com (X.Z.); junxingzhang163@163.com (J.Z.); mayun_666@126.com (Y.M.); 2 Institute of Animal Science and Veterinary Medicine, Shandong Academy of Agricultural Sciences, No. 23788, Gongyebei Road, Jinan 250100, China; liyanqin@sdox.cn; 3 Shandong Ox Livestock Breeding Co., Ltd., Jinan 250100, China; 4 Laboratory of Animal Genetics, Breeding and Reproduction, Ministry of Agriculture of China, National Engineering Laboratory of Animal Breeding, College of Animal Science and Technology, China Agricultural University, Beijing 100193, China; luohanpeng@cau.edu.cn (H.L.); luhaibo979@163.com (H.L.); 13369452684@163.com (L.M.); doujinhuan_cau@163.com (J.D.); 5 Beijing Consortium for Innovative Bio-Breeding, Beijing 101206, China

**Keywords:** antibody-mediated immune response, RNA-seq, transcriptome analysis, dairy bulls

## Abstract

**Simple Summary:**

Genetic selection for immune response features may be a useful strategy for enhancing animal health. In this study, we identified the DEGs for dairy bulls with high- and low-AMIR. Results revealed that several DEGs were substantially associated with the regulation of locomotion, tissue development, immune response, and detoxification. In addition, the results of the KEGG pathway analysis showed that most DEGs were enriched in pathways related to disease, inflammation, and immune response, providing insights for genomic selection. These findings help to better comprehend the immune response mechanism in dairy bulls.

**Abstract:**

Enhancing the immune response through breeding is regarded as an effective strategy for improving animal health, as dairy cattle identified as high immune responders are reported to have a decreased prevalence of economically significant diseases. The identification of differentially expressed genes (DEGs) associated with immune responses might be an effective tool for breeding healthy dairy cattle. In this study, antibody-mediated immune responses (AMIRs) were induced by the immunization of hen egg white lysozyme (HEWL) in six Chinese Holstein dairy bulls divided into high- and low-AMIR groups based on their HEWL antibody level. Then, RNA-seq was applied to explore the transcriptome of peripheral whole blood between the two comparison groups. As a result, several major upregulated and downregulated genes were identified and attributed to the regulation of locomotion, tissue development, immune response, and detoxification. In addition, the result of the KEGG pathway analysis revealed that most DEGs were enriched in pathways related to disease, inflammation, and immune response, including antigen processing and presentation, Staphylococcus aureus infection, intestinal immune network for IgA production, cytokine–cytokine receptor interaction, and complement and coagulation cascades. Moreover, six genes (*BOLA-DQA5*, *C5*, *CXCL2*, *HBA*, *LTF*, and *COL1A1*) were validated using RT-qPCR, which may provide information for genomic selection in breeding programs. These results broaden the knowledge of the immune response mechanism in dairy bulls, which has strong implications for breeding cattle with an enhanced AMIR.

## 1. Introduction

Disease occurrence remains a prevalent problem, causing substantial financial losses and problems with animal welfare, as ethical issues with antibiotic use are highly concerning [1]. Furthermore, a decline in genetic trends for reproductive and health traits has been observed since milk production traits have played a dominant role in selection programs of dairy cattle for many decades, and genetic selection for improving production traits has negatively impacted overall animal health and well-being [2,3]. Therefore, improving the overall health of cows through genetic improvement may be an important route to reducing farm costs and increasing profits, and genetic selection for health traits to breed dairy cattle with high disease resistance has been carried out. Six direct health traits, including resistance to ketosis, mastitis, hypocalcemia or milk fever, metritis, retained placenta, and misplaced abomasum, were added to the U.S. genomic evaluation system in 2018 [4]. However, health traits generally have low heritability (<0.05) [5], a non-normal distribution of phenotype data, and subjectivity in diagnosis. In addition, selection for resistance to a specific disease may leave individuals susceptible to other diseases because the immune response needed to control different pathogens may have antagonistic genetic relationships [6]. Therefore, this makes the selection of the overall immune response appealing.

The immune response—the ability to resist infectious diseases—is a complex trait for the dairy industry. Animals tend to mount a cell-mediated immune response (CMIR) in response to intracellular pathogens and an antibody-mediated immune response (AMIR) in response to extracellular pathogens [6]. CMIR and AMIR can be evaluated by delayed-type hypersensitivity (DTH) and modified ELISA after immunization with antigens mentioned by Thompson-Crispi et al. [6]. Furthermore, previous studies have found that adaptive immune response traits can be inherited, with heritability of CMIR traits of 0.19–0.43 and AMIR traits of 0.16–0.41, depending on time and antibody isotype [6,7]. Immune response heritability is comparable to that observed for milk production traits and significantly greater than estimates for specific clinical or subclinical disease resistance [8], indicating that selection for immune response is expected to result in large genetic gains. In addition, Holstein cows with different immune response phenotypes do not differ significantly in production variables such as milk output, milk fat, or milk protein; multiple studies have found an association between the antibody response and the incidence of mastitis [8,9,10,11,12]; a positive correlation between the immune response and reproductive and longevity traits has been reported [6,10]. Therefore, breeding programs for immune response traits could improve the health of dairy cows, without negative impact on milk production, longevity, or reproductive traits.

For dairy cattle breeding programs, with the invention and advancement of molecular quantitative genetics, identifying genes underlying immune responses and incorporating them into genetic evaluation systems would be beneficial. High-throughput RNA-sequencing (RNA-Seq) technology has emerged as a popular method for detecting differentially expressed genes (DEGs) across different biological conditions. By adding biological information from DEGs, the accuracy of genomic prediction based on the best linear unbiased prediction (GFBLUP) model for mastitis and milk production traits in dairy cattle was improved [11]. It has also been applied to analyze differences in certain disease susceptibility or resistance, including ketosis and mastitis [12,13]. In other studies, RNA-Seq transcriptome analysis has successfully revealed up- and downregulated genes relevant to the host immune response during viral infections, including ovine and goat bluetongue virus [14], bovine papillomatosis [15], and bovine viral diarrhea virus (BVDV) type I [16]. However, of these, limited studies on the transcriptome of the bovine general immune response have been reported, especially for dairy bulls.

Therefore, identifying DEGs related to immune responses is crucial for explaining and understanding genetic variations in disease prevention and diagnosis [17,18] and may help improve the immunity of animals. In this study, utilizing the whole blood transcriptome of dairy bulls classified as high- and low-AMIR groups, we aimed to (1) identify DEGs associated with the adaptive immune response in cattle; (2) study the key signaling pathways of the immune response and the changes of various regulatory molecules, thus elucidating the immunomodulatory mechanisms related to disease resistance in dairy cattle and providing new insights for improving animal health and welfare from an immunological perspective.

## 2. Materials and Methods

All procedures involving experimental animals were approved by the Animal Welfare Committee of Ningxia University (NXUC20210801). All efforts were made to reduce the suffering and discomfort of experimental animals.

### 2.1. Experimental Animals and Immunization Protocol

**Figure 1:**
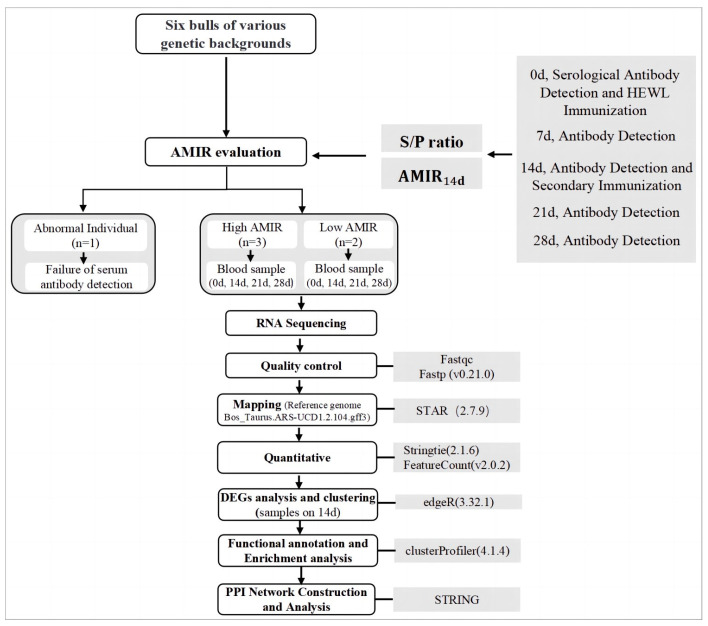
Workflow of this study.

We randomly selected six healthy dairy bulls aged between 4.9–6.7 years and weighing 1000–1100 kg for this study. The six bulls were Holstein breed with similar genetic merit. They were of various genetic backgrounds, according to the pedigree information. Additionally, principal component analysis (PCA) was carried out to analyze the genetic relationships among these animals based on genotypes, showing no significant clustering according to Supplementary Materials File S1: Figure S1. All experimental animals received no veterinary treatment during the course of the experiment. Immunization protocols were conducted from September 2021 to October 2021, and AMIR indicators were evaluated by modified ELISA based on a previous study [6]. On d0 and d14, the bulls received an intramuscular injection of 0.5 mg of hen egg white lysozyme (HEWL; Sigma L4919, Shanghai, China), 0.5 mg heat-killed *Candida albicans* (HKCA-#tlrl-hkca, InvivoGen, San Diego, USA), and 0.5 mg of Quil-A adjuvant (#vacquil, InvivoGen, Toulouse, France) dissolved in 1 mL of phosphate-buffered saline (PBS, pH 7.4). On both sides of the neck, an intramuscular injection of 1 mL was given using a single-use needle. The workflow of this study is summarized in Figure 1.

### 2.2. HEWL Serum Antibody Detection

Blood was collected via jugular venipuncture on d0, d7, d14, d21, and d28 to evaluate serum antibodies to the HEWL. Fetal calf serum served as the negative control, and d21 sera collected after two immunizations served as the positive control. Flat-bottomed 96-well polystyrene plates were coated with 0.05 mg/mL HEWL (Sigma L4919) in CoatingBuffer (0.05 M sodium bicarbonate, pH 9.6). We added 100 μL of diluted coating antibody to each well and incubated it at 4 °C overnight. The coating protein was removed, and the plates were washed five times using 200 μL of ELISA wash solution (50 mM Tris, 0.14 M NaCl, 0.05% Tween 20, pH 8.0). The plates were then blocked with blocking solution (50 mM Tris, 0.14 M NaCl, 0.05% Tween 20, pH 8.0) for 1 h at 37 °C and washed five times again. Sera were diluted in sample/conjugate diluent (50 mM Tris, 0.14 M NaCl, 0.05% Tween 20, pH 8.0) at 1/50 and 1/200 and incubated for 1 h at 37 °C. The plates were washed five times, and the secondary antibody was dissolved in sample/conjugate diluent and incubated at room temperature for 0.5 h followed by five washes; we used HRP-conjugated sheep anti-bovine IgG1 detection antibody (Bethyl Laboratories Inc., Montgomery, AL, USA, Bovine IgG1 ELISA Quantitative Set, Cat. No. E10-116). A total of 100 μL of TMB Substrate Solution was added to each well. The reaction was stopped by adding 100 μL of Stop Solution to each well after the plate had been developed for 15 min in the dark at room temperature. Antibody concentration was measured with an automatic ELISA microplate reader set to an absorbance of 450 nm and 630 nm and expressed in optical density units (OD). The sample to positive (S/P) ratio [6,19] and AMIR_14d_ (the ratio of optical density values at d14 of the immunization protocol to those at d0) were used to determine the AMIR based on the phenotypic deviation of serum antibody responses, and the formula is shown below. Accordingly, experimental bulls were classified into high- and low-AMIR groups.

$$\frac{S}{P}=\frac{{OD}_{14d}-Negative\;Control\;O.D.value}{Positive\;Control\;O.D.value-Negative\;Control\;O.D.value}$$

$${AMIR}_{14d}=\frac{{OD}_{14d}}{{OD}_{0d}}$$

### 2.3. RNA-Seq and Transcriptome Quantification

Given that the test animals produced a very small amount of antibodies on d7, transcriptome analysis was not performed on blood samples at this time point. From the blood samples collected on d0, d14, d21, and d28 of the immunization protocol by PAXgene Blood RNA tubes, total RNA was stabilized and extracted as directed by the manufacturer. RNA integrity was determined on a Bioanalyzer 2100 (Agilent Technologies, CA, USA) by a Nano 6000 Assay Kit with an acceptable RIN value above 8.0. Total RNA was used for the library construction following the Illumina NEB Next Ultra™ RNA protocol. The Agilent Bioanalyzer 2100 system was used to evaluate the library’s quality. According to the manufacturer’s instructions, the TruSeq PE Cluster Kit v3-cBot-HS (Illumina, San Diego, CA, USA) was used to cluster the index-coded sample data on a cBot Cluster Generation System. The library preparations were sequenced on an Illumina Novaseq platform after cluster generation, producing 150 bp paired-end reads.

Fast QC software (v0.11.9, http://www.bioinformatics.babraham.ac.uk/projects/fastqc/, accessed on 25 February 2022) was used to assess the quality of the sequencing reads, and Fastp (v0.21.0) was used for global trimming [20]. The clean, high-quality data served as the basis for all downstream studies. Clean reads were indexed to the reference (*Bos taurus*) cattle genome ARS-UCD1.2 and mapped using STAR (2.7.9) [21]. Thereafter, StringTie (2.1.6) (https://ccb.jhu.edu/software/stringtie/, accessed on 5 March 2022) was used to assemble the obtained mapped reads. FeatureCount (v2.0.2) was used to count the number of reads mapped to each gene [22].

### 2.4. Analysis of DEGs

The identification of DEGs between high- and low-AMIR groups was based on the expression level of each transcript. An exact test based on quantile-adjusted conditional maximum likelihood (qCML) was completed for DEG screening using the edgeR R package [23]. The threshold, fold change ≥2 and <0.05 for the alpha of the false discovery rate (FDR), was set according to a previous transcriptome study [24,25]. Furthermore, the similarity level of each sample was determined using hierarchical clustering of DEGs.

To generalize the functions and pathways of DEGs, the sequences were functionally annotated and classified by comparing them to the gene ontology (GO) and the Kyoto Encyclopedia of Genes and Genomes (KEGG) databases. By using the “clusterProfiler” package [26], which corrects for gene length bias, KEGG pathways and GO terms were enriched. DEGs with a *p*-value less than 0.05 were found to be highly enriched in GO terms and KEGG pathways.

The STRING (http://string-db.org/, accessed on 20 March 2023) database was used to investigate a PPI network among the DEGs to better comprehend and foresee the biological activity of the detected DEGs based on GO and KEGG pathway enrichment analysis. The cutoff threshold was set at a confidence score > 0.7 and a *p* < 0.05.

The RNA-Seq data were confirmed using RT-qPCR on six randomly selected genes. Primer 5.0 was utilized to design the primers, and General Biosystems Co., Ltd. (Anhui, China) was employed to synthesize the primers. Total RNA was reversed to cDNA by the TaKaRa Reverse Transcription Kit (TaKaRa, Dalian, China) according to the instructions provided by the manufacturer. Quantitative PCR was carried out on a 7500 Fast Real-Time PCR System (Thermo Fisher Scientific, Waltham, MA, USA) with the TB Green^®^ Premix Ex TaqTM Kit (TaKaRa). GAPDH was selected as an internal control. The2^−ΔΔCT^ method was used to examine all data from three biological replicates for each sample [27].

### 2.5. Gene Expression Pattern Profiling

Fifty-six genes with significant differences between the high- and low-AMIR groups were selected for time series analysis. These genes are related to immune response, including the BOLA family, the CXCL chemokine family, the hemoglobin family, the interleukin family, the tumor necrosis factor family, etc. The Mfuzz R package’s fuzzy c-means algorithm was used to profile DEGs based on their expression patterns. For each time point (d0, d14, d21, and d28) of the immunization protocol, the average FPKM value for each gene was utilized as the input. Following standardization, each gene was assigned to a distinct cluster based on its membership value.

## 3. Results

### 3.1. Experimental Animals and Grouping

**Figure 2:**
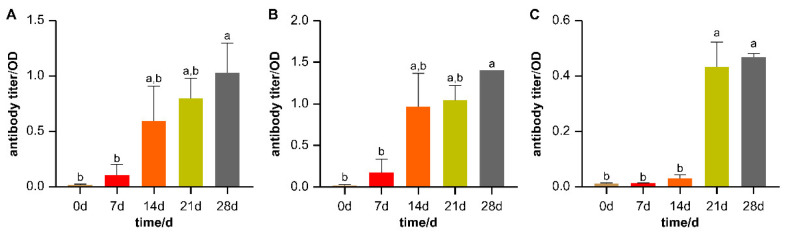
HEWL antibody titers measured by ELISA on days 0, 7, 14, 21, and 28. The mean and standard error are shown. Means without a common letter differ significantly (*p* < 0.05). (**A**) Trend of changes in the titer of antibody against HEWL in all experimental animals; (**B**) trend of changes in the titer of antibody against HEWL in the high-AMIR group; (**C**) trend of changes in the titer of antibody against HEWL in the low-AMIR group.

The obtained HEWL antibody titers on d0, d7, d14, d21, and d28 of immunization are shown in Figure 2A. The antibody response showed a gradually increasing trend within 21 days of immunization. The initial antibody titer was close to zero in the peripheral blood, indicating that the selection of experimental animals was reasonable. After d7 immunization, only a slight increase in the antibody response was induced to the HEWL immunogen. Subsequently, more antibodies were produced from d14. The antibody titer was highest at d28 and was significantly higher than that on d0 and d7 (*p* < 0.05).

The six animals were separated into high- and low-AMIR groups based on the S/P ratio and AMIR14d. The two evaluation criteria obtained similar results. For the high-AMIR group, there were three identified animals, H1, H2, and H3, whereas two animals, L1 and L2, were part of the low-AMIR group. The sixth individual was discarded because the serum antibody detection was not successful.

**Figure 3:**
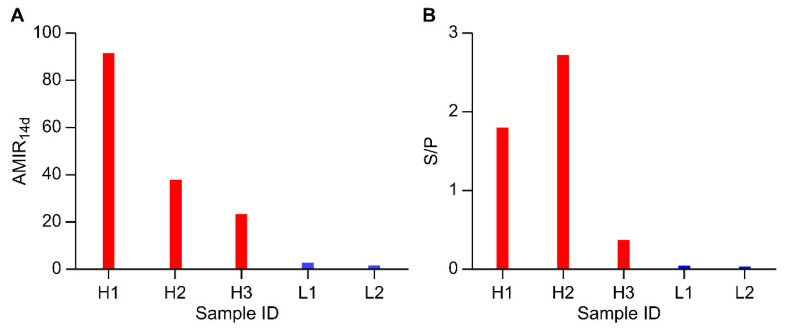
The level of immune response in experimental animals based on the phenotypic deviation of the serum antibody response. (**A**) The AMIR14d value is the ratio of optical density values at d14 of the immunization protocol to those at d0; (**B**) sample-to-positive (S/P) ratio of HEWL antibody level from serum IgG1 at d14 of the immunization protocol. Red pillars represent high-AMIR animals, and blue pillars represent low-AMIR animals.

We also identified the trend of changes in the titer of antibody against HEWL in the two groups (Figure 2B,C). There was an upward trend in both groups. In the high-AMIR group, except for the significantly higher antibody titer on d28 compared to d0 and d7, there were no significant differences among samples at any other time points, and the antibody titer on d14 reached a relatively high level (Figure 2B). In the low-AMIR group, the antibody titers on d21 and d28 were remarkably higher than any other time points, and the antibody titer at d14 remained at a low level (Figure 2C). The AMIR14d values and S/P of each sample are shown in Figure 3. The two indicators, AMIR_14d_ and S/P, for animals in the high-AMIR group were significantly higher than those of the low-AMIR group.

### 3.2. Differentially Expressed Genes

For each sample, paired-end transcriptome sequencing produced at least 6.0 Gbp of raw data. According to the trimmed results, the Q30 (%) of clean data for all samples collected on d14 was greater than 93.87%, and the GC contents ranged from 52.40 to 53.22% (Table 1). Approximately 89.97–93.12% of clean reads were uniquely aligned to the reference genome. A detailed description of the RNA-seq data for blood samples on d0, d21, and d28 is provided in Supplementary Materials File S1: Table S1.

**Figure 4:**
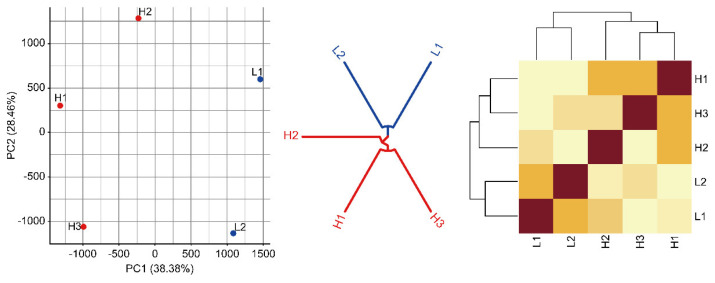
Cluster of high-AMIR and low-AMIR based on read counts. PCA, cluster dendrogram, and correlation matrix were performed for blood samples collected at d14 of the immunization protocol. Red points in the dot plot represent high-AMIR samples. Blue points in the dot plot represent low-AMIR samples.

PCA and cluster analysis were performed on the gene expression levels of blood samples on d0, d14, d21, and d28 of the immunization protocol. The clustering results of blood samples on d0 were scattered and had no obvious regularity (Supplementary Materials File S1: Figure S2). The high- and low-AMIR groups differed significantly in RNA-seq counts for blood samples collected on d14 of the immunization protocol based on PCA and clustering structure (Figure 4). According to the cluster results of blood samples on d21 and d28 of immunization (Supplementary Materials File S1: Figures S3 and S4), samples could not be clustered regularly. Thus, blood samples from the high- and low-AMIR groups on d14 were used for DEG analysis.

**Figure 5:**
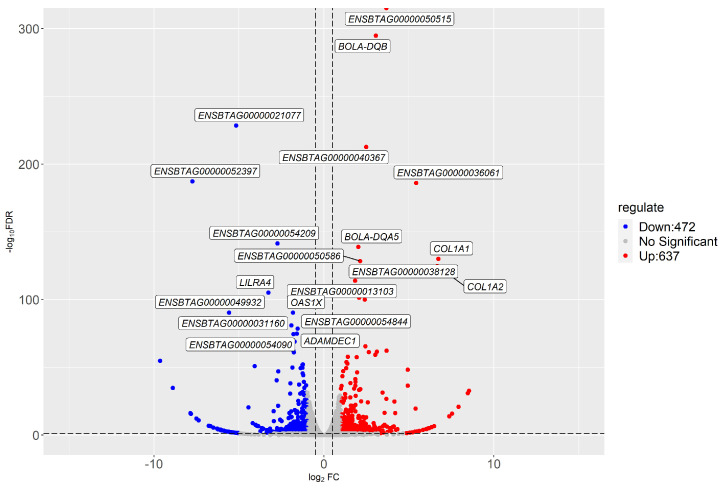
Volcano plot displaying significantly expressed genes in the comparison group, low-AMIR vs. high-AMIR. The red and green dots represent genes that have been significantly upregulated or downregulated, respectively.

DEGs were identified using the qCML method. Based on the threshold level (fold change ≥ 2 and FDR < 0.05), 1109 genes were identified as differentially expressed (Figure 5). Compared with the high-AMIR group, 637 genes were upregulated, while 472 genes were downregulated in the low-AMIR group.

### 3.3. Functional Enrichment Analysis

**Figure 6:**
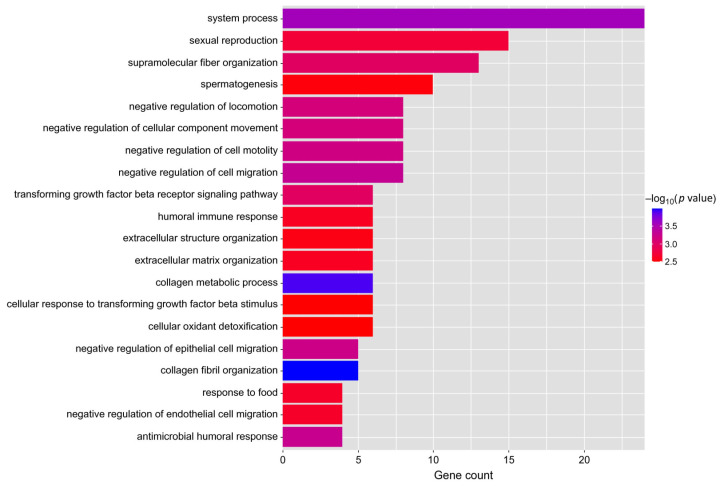
Analysis of DEGs using gene ontology (GO) functional enrichment. Only the top 20 biological processes are mentioned.

GO analysis of 1109 DEGs showed that 179 significant GO items were enriched (*p* < 0.05), including 127 biological processes (GO-BP), 32 cellular components (GO-CC), and 20 molecular functions (GO-MF) (Supplementary Materials File S2). In GO-BP terms, DEGs in the comparison groups low-AMIR vs. high-AMIR were primarily enriched in those related to regulation of locomotion (*DCN*, *JUP*, *IGFBP3*, *ANGPT2*, and *COL3A1*), tissue development (*COL1A1* and *COL1A2*), immune response (*LTF*, *PGLYRP1*, *LPO*, *C5, SERPING1,* and *RARRES2*), and detoxification (*HBM*, *HBE2*, and *HBA*). Of all GO-MF terms, four GO terms were related to receptor activity, namely receptor ligand activity, signaling receptor activator activity, receptor regulator activity, and signaling receptor binding. The key genes enriched in GO-MF terms were *TGFBP2*, *IL34*, *STC1*, *INHA*, *CXCL2*, *CCK,* and *GRO1* (Supplementary Materials File S2). The main purpose of our research is to explore biological functions, so we focus on biological processes, and the top 20 significant GO-BP terms are shown in Figure 6.

**Figure 7:**
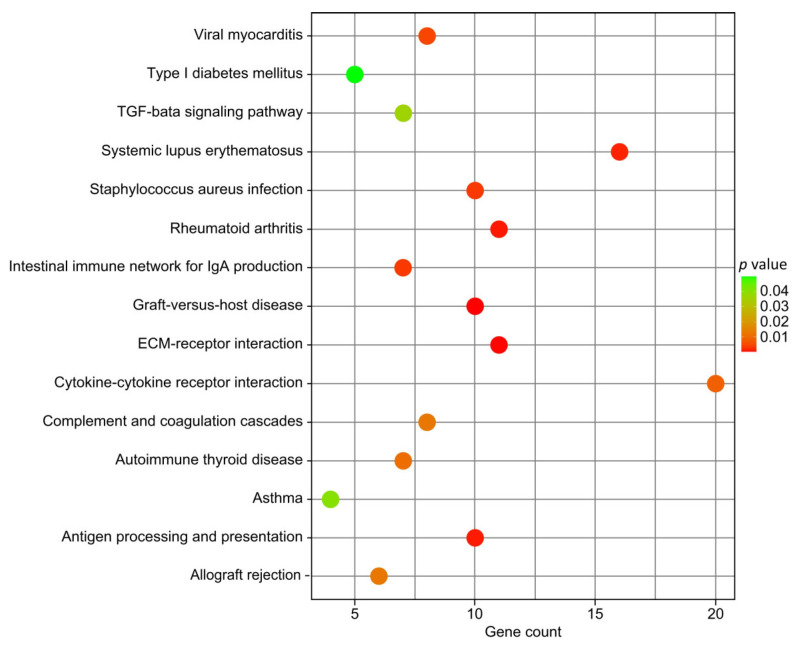
The main enriched Kyoto Encyclopedia of Genes and Genomes (KEGG) pathways for DEGs. Count: number of genes in each pathway.

The KEGG enrichment analysis of DEGs identified 42 highly enriched pathways, mostly related to disease, inflammation, and immune response. Sixteen of all significantly enriched pathways (*p* < 0.05) were associated with various diseases. Graft-versus-host disease, ECM–receptor interaction, antigen processing and presentation, Staphylococcus aureus infection, intestinal immune network for IgA production, viral myocarditis, cytokine–cytokine receptor interaction, autoimmune thyroid disease, complement and coagulation cascades, allograft rejection, and TGF-beta signaling pathways were found to be enriched in canonical pathways related to immune response and inflammation (Figure 7, Table 2, and Supplementary Materials File S3). The main upregulated genes enriched in KEGG included *BOLA-DQB*, *BOLA-DQA5*, *COL1A1*, *COL1A2*, *C5*, *KIR3DL1*, *CXCL12*, *CCR10*, *IL36A*, *IL1RL2*, and *IL34*, while *TGFB2*, *ITGB4*, *KIR3DL2*, *GRO1*, *CXCL2*, and *JSP.1* were downregulated. GO and KEGG analyses revealed that several DEGs were involved in the AMIR.

**Figure 8:**
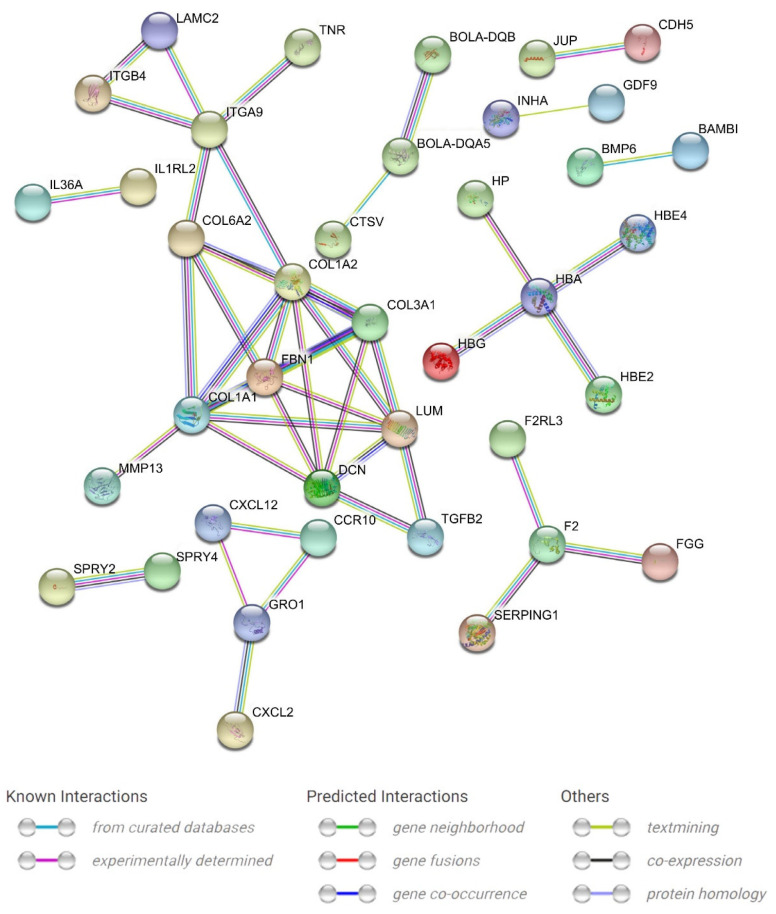
A protein–protein interaction (PPI) network construction based on the STRING database. The interaction was carried out with a confidence score of 0.7. Nodes represent genes, and lines between nodes refer to edges indicating various types of interactions, which are denoted by different colors and defined by the legends in the figure; stand-alone nodes without edges were removed.

STRING analysis was performed to investigate the DEG potential interaction network (Figure 8). Among the upregulated genes, *DCN*, *COL1A1*, *COL1A2*, *COL3A1*, *HBA, F2,* and *BOLA-DQA5* were situated in the network’s core and connected to many other DEGs; for the downregulated genes, the key points included *FBN1* and *ITGA9*, which linked to more genes. Additionally, not all DEGs displayed a link with others because their functions were either unclear or unrelated to one another, which were not included in the analysis.

**Figure 9:**
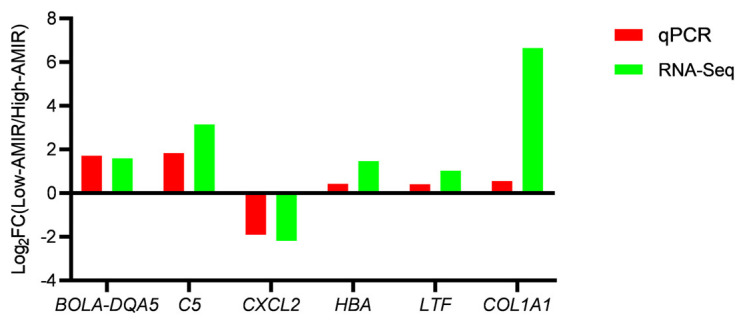
Validation of RNA-Seq results using qPCR. The qPCR and RNA-Seq data bars are colored red and green, respectively.

RT-qPCR was performed to validate the gene expression identified by RNA-seq. The six genes selected were from various gene families. Thus, the relationship between each gene family and the immune response was further confirmed. Additionally, the results demonstrated that the expression levels of the six genes generally agreed with the RNA-Seq results based on read counts (Figure 9).

### 3.4. Expression Pattern of Selected DEGs

**Figure 10:**
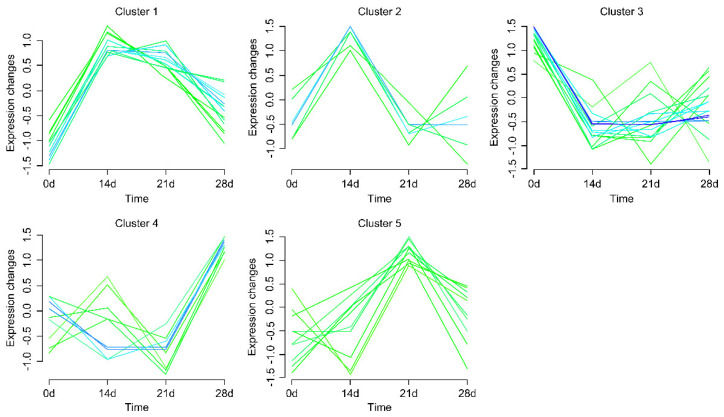
Gene clusters with similar expression patterns. The ‘Mfuzz’ R package was used to analyze DEG expression patterns. Based on the similarity of expression patterns, five clusters were identified.

Soft clustering was performed on the expression of the selected DEGs at different time points for the high-AMIR individuals. Since there were only a few genes, the number of clusters was manually set in this study. Finally, all genes were classified into five clusters. As shown in Figure 10, the expression of genes in Cluster 1 (*IL5RA*, *RARRES2*, *F2*, *SPRY4,* and *ITGB4*) increased after the immunization, maintaining a high level between d14 and d21, and then decreased slowly. The genes in Cluster 2 (*IL34* and *CCK*) reached peak expression at d14 and then declined. In Cluster 3 (*LTF*, *PGLYRP1*, *ANGPT2*, *C5*, *BOLA-DQA5*, *BOLA-DQB*, *HBA*, *HBE2*, *HBE4*, *HBM*, *HBG,* and *CXCL2*), genes were downregulated from d0 to d14 of the immunization protocol, subsequently remaining relatively stable. The genes in Cluster 4 (*SERPING1* and *IL36A*) showed irregular changes before d21, but were then upregulated. The expression pattern of genes in Cluster 5 (*IL1RL2*, *COL1A1*, and *DCN*) reached a peak at d21 after immunization. The gene list from each cluster and the FPKM values of each gene at different time points are presented in Supplementary Materials File S4. Of the six genes that were selected for RT-qPCR, *BOLA-DQA5*, *C5*, *CXCL2*, *HBA*, and *LTF* represented Cluster 3, while *COL1A1* represented Cluster 5.

## 4. Discussion

### 4.1. Experimental Design and Samples Information

Research has suggested that genetic improvement is dominated by the sire-daughter path compared to the dam-daughter path [28]. Another study also supports the argument that the sire has a more significant effect on the genetic improvement of dairy herds than the dam [29], and high immune response sires reduce the disease incidence in large commercial dairy populations in North America [30]. Likewise, the Immunity+™ sire line, which was identified with superior immune responsiveness by the Semex Alliance utilizing HIR Technology, may be expected to produce daughters with superior immunity or disease resistance [10]. Therefore, Holstein bulls as mature sires were selected as research subjects in the present study to contribute knowledge to the selection process of high-resistance breeding bulls.

Although there were only a few experimental animals, the results are encouraging and could be used to support future research based on a larger population. Because dairy cattle are bred by artificial insemination, there are a relatively small number of dairy bulls. Some bulls, especially Holstein bulls, may be closely related, which increased the difficulty of sample selection. In order to increase the reliability of the test results, six Holstein bulls were initially used to perform the experiment. Unfortunately, one bull sample was discarded due to uncertainty. However, serum antibody levels in the three high-AMIR animals were significantly higher than those in the low-AMIR group (Figure 3), indicating that the experimental animals were representative. Additionally, the selection of mature sires that have produced offspring may facilitate subsequent validation in the cow population. In addition, we referred to a published paper that also used Holstein dairy cattle as experimental animals, but only five samples were analyzed [31].

Given the advantage that the RNA-Seq technique can detect a higher proportion of DEGs than gene chips, it has been applied to map key genes for susceptibility or resistance to certain diseases, including ketosis and mastitis [12,13]. Instead of specific disease resistance, the current study, for the first time, reported RNA-Seq results for overall disease resistance and the adaptive immune response in dairy bulls. The ELISA results revealed that all animals showed an almost similar antibody titer, which was close to zero at d0. After the same treatment protocol, each individual produced an antibody reaction, and there was a significant difference in S/P ratio or AMIR14d based on antibody titer at d14 between the two groups. Therefore, the DEGs found in the transcriptome study were mostly attributed to the immune response of the animals to the HEWL immunogen. The method suggested here could detect differential gene expression profiles that may be associated with general disease resistance.

### 4.2. Differential Genes Screening and Functional Enrichment

The study of GO functional enrichment indicated upregulated DEGs related to the regulation of cell motility, tissue development, humoral immune response, and detoxification. *LTF*, *PGLYRP1*, *C5*, *SERPING1*, and *RARRES2* were significantly enriched in the items related to immune response, namely the humoral immune response and antimicrobial humoral response. Lactotransferrin (*LTF*) is a gene in the transferrin family whose protein product is found in neutrophil secondary granules. It can regulate macrophage activity and stimulate lymphocyte synthesis by binding macrophages and lymphocytes, promoting lymphocyte proliferation by inducing specific immune responses. It has been proposed as a potential biomarker of mastitis susceptibility/resistance [32], subclinical endometritis (SCE) [33], and Mycobacterium avium subspecies—a paratuberculosis infection in dairy cattle [34]. Bovine *PGLYRP1*, found in naive neutrophil dense/large granules and neutrophil phagolysosomes [35], was the first member of PGRP identified for its bactericidal activity in vitro. Polymorphism in the gene was shown to be associated with mastitis resistance, and the H3H3 haplotype was regarded as a valuable genetic indicator of combination haplotypes for developing mastitis resistance in the Chinese Holstein [36]. Both *C5* and *SERPING1* are involved in the regulation of the complement system. During complement activation, C5 breaks down to produce C5a, which is a major allergic toxin involved in neutrophil chemotaxis and the release of pro-inflammatory cytokines [37]. SERPING1 encodes the C1 inhibitor, which is important in inhibiting complement component 1 (C1) and may be associated with the conventional complement activation pathway [38]. Chemerin (RARRES2) is an endogenous leukocyte chemoattractant and is frequently downregulated in several tumor types when compared to normal tissue [39]. This corresponds to the downregulated *RARRES2* gene expression in low responders found in the present study.

DEGs associated with detoxification were mainly members of the hemoglobin (Hb) family, including *HBE4*, *HBG*, *HBM*, *HBE2*, and *HBA*. Long regarded as inert oxygen carriers, red blood cells are emerging as potential modulators of the innate immune response [40]. Hb and heme reportedly have immunomodulatory effects and could produce antimicrobial reactive oxygen species (ROS) to defend against invading microorganisms [40]. A recent study also indicated that Hb had immunomodulatory, signal transduction, and bactericidal effects [41]. Moreover, there is growing evidence that Hb is not only an oxygen transporter but also contributes substantially to the innate immune system. Bovine Hb is an animal protein that has been reported as a source of bioactive peptides. Nedjar-arroume et al. [42] extracted and discovered four antimicrobial peptides from bovine Hb that displayed antibacterial activity against *Micrococcus luteus A270*, *Listeria innocua*, *Escherichia coli,* and *Salmonella enteritidis*. In the present study, Hb family genes were considerably enriched in GO terms related to detoxification, further confirming the important role of Hb in immune regulation.

Among the KEGG pathways enriched in this study, nine pathways were consistent with the results obtained by the GWAS study conducted to assess overall adaptive immune response in cattle [43]; these included Graft-versus-host disease, antigen processing and presentation, systemic lupus erythematosus, intestinal immune network for IgA production, viral myocarditis, autoimmune thyroid disease, allograft rejection, asthma, and type I diabetes mellitus. In one study, the relationship between immune response and mastitis was examined, and it was found that high-AMIR cows had a significantly decreased incidence of clinical mastitis compared to average and low-AMIR cows [8]. Meanwhile, S. aureus has been considered one of the most important mastitis pathogens [44,45]. In the present study, S. aureus infection was identified as a KEGG pathway significantly related to the AMIR, in agreement with the research above.

The cytokine–cytokine receptor interaction is critical to health during immune and inflammatory responses to diseases [46]. In the present study, many DEGs were found to be enriched in the cytokine–cytokine receptor interaction pathway, including the interleukin family and CXC chemokine family genes. In addition, *IL-34* expression has been shown to be dysregulated in a variety of immune-inflammatory diseases, infections, and metabolic and neurological disorders [47]. It has also been noted that there is a correlation between changes in IL-34 content and disease parameters in a variety of pathological conditions, including rheumatoid arthritis, systemic lupus erythematosus, heart failure, and viral infections. Genome-wide association analysis on cows showed that *IL-34* was significantly associated with both clinical mastitis and SCS [48]. Also, the IL-34 level has been viewed as a helpful biomarker for predicting the progression of disease. CXCL2 is an inflammatory factor that directly participates in biological processes such as immune regulation, inflammatory responses, and angiogenesis by binding to the G protein-coupled receptor CXCR. Sharifi et al. [49] showed that CXCL2 could be used to distinguish mastitis from healthy samples with an accuracy of 83.85%.

Bovine lymphocyte antigen (*BOLA*) genes were also significantly enriched in multiple pathways, according to the KEGG pathway enrichment analysis. Known as the major histocompatibility complex (MHC) of cattle, BOLA genes are located on chromosome 23 in cattle [43] and have been intensively investigated by researchers because of their antigen presentation function. In addition, polymorphism in BOLA genes was found to be closely associated with bovine disease resistance and immunological traits [50]. According to the structure and function of the encoding products, BOLA genes can be classified into three categories: class I, class II, and class III. Among the DEGs identified significantly by KEGG analysis in the present study, *BOLA-DQA5* and *BOLA-DQB* are class II genes. In cattle, polymorphism in class IIa genes affects the magnitude and epitope specificity of antigen-specific T-cell responses to FMD virus peptides. As indicated in Figure 5, the expression levels of *BOLA-DQA5* and *BOLA-DQB* genes were extremely significantly different between high- and low-AMIR groups, confirming the importance of this gene in immune regulation. This is in accordance with previous studies.

The incorporation of DEGs from this study into animal genetic improvement programs such as selective breeding and genetic manipulation might improve cattle immunity.

## 5. Conclusions

In the present study, several critical DEGs were found to be associated with the high and low general AMIRs in dairy bulls, of which, six important genes (*BOLA-DQA5*, *C5*, *CXCL2*, *HBA*, *LTF*, and *COL1A1*) were validated by RT-qPCR. The DEGs, GO terms, and KEGG pathways identified in this study could help in further exploration of the underlying mechanisms of immune responses in dairy cattle. This work addressed a knowledge gap in the transcriptome differential analysis in the peripheral blood transcriptome of high- and low-AMIR bulls, and the identification of key genes could provide useful information for the breeding of enhanced immune response sires.

## Figures and Tables

**Table 1:** An overview of RNA-Seq data for each sample on d14.

Sample ID	Raw Reads	Clean Reads	Clean Bases	Error Rate (%)	Q30 (%)	GC Content (%)	Uniquely Mapping Rate (%)
H1	46,711,716	46,104,046	6.18 G	0.03	94.26	52.74	92.96%
H2	41,180,040	40,655,298	5.45 G	0.03	93.87	52.40	92.71%
H3	45,339,678	44,767,294	6.0 G	0.03	93.80	52.73	93.12%
L1	41,720,406	41,196,110	5.51 G	0.03	94.38	53.22	89.97%
L2	47,547,798	46,913,208	6.29 G	0.03	93.45	52.57	92.52%

**Table 2:** KEGG pathways substantially enriched in immune response and inflammation.

Pathway	ID	*p*-Value	Upregulated Genes	Downregulated Genes
Graft-versus-host disease	bta05332	6.14 × 10^−5^	*BOLA-DQB, BOLA-DQA5, KIR3DL1, ENSBTAG00000000966, ENSBTAG00000049367, ENSBTAG00000052514*	*JSP.1, KIR3DL2, ENSBTAG00000039813, ENSBTAG00000049260*
ECM–receptor interaction	bta04512	0.000177	*COL1A1, COL1A2, COL6A1, COL6A2, GP5, LAMA4, LAMC2, SV2C*	*ITGA9, ITGB4, TNR*
Antigen processing and presentation	bta04612	0.000895	*ENSBTAG00000000966, ENSBTAG00000049367, ENSBTAG00000052514, BOLA-DQA5, BOLA-DQB, CTSV, KIR3DL1*	*ENSBTAG00000049260, JSP.1, KIR3DL2*
Systemic lupus erythematosus	bta05322	0.001285	*ENSBTAG00000006864, ENSBTAG00000048268,* *BOLA-DQA5, BOLA-DQB, C5, H2AC8*	*ENSBTAG00000038433, ENSBTAG00000049260, H2BC13, H2BU1*
*Staphylococcus aureus* infection	bta05150	0.003058	*ENSBTAG00000006864, ENSBTAG00000048268, BOLA-DQA5, BOLA-DQB, C5, FGG, KRT18, KRT26*	*ENSBTAG00000049260, KRT23*
Intestinal immune network for IgA production	bta04672	0.003073	*ENSBTAG00000048268, BOLA-DQA5, BOLA-QB, CCR10, CXCL12, TNFRSF17*	*ENSBTAG00000049260*
Viral myocarditis	bta05416	0.004436	*MYH7, BOLA-DQB, BOLA-DQA5, MYH6, ENSBTAG00000048268*	*JSP.1, CYCT, ENSBTAG00000049260*
Cytokine–cytokine receptor interaction	bta04060	0.008272	*CCR10, CX3CL1, CXCL12, GH1, IL1RL2, IL34, IL36A, INHA, MPL, TNFRSF17, TNFSF15, ENSBTAG00000052397*	*BMP6, CCL19, CXCL2, GDF9, GRO1, IL5RA, TGFB2, TNFSF4*
Autoimmune thyroid disease	bta05320	0.010892	*TSHB, BOLA-DQB, BOLA-DQA5, ENSBTAG00000048268*	*JSP.1, ENSBTAG00000039813, ENSBTAG00000049260*
Complement and coagulation cascades	bta04610	0.01253	*C5, F2, F2RL3, FGG, SERPING1, ENSBTAG00000006864, ENSBTAG00000023026*	*PROS1*
Allograft rejection	bta05330	0.012633	*BOLA-DQB, BOLA-DQA5, ENSBTAG00000048268*	*JSP.1, ENSBTAG00000039813, ENSBTAG00000049260*
TGF-beta signaling pathway	bta04350	0.036167	*ENSBTAG00000005140, DCN*	*FBN1, BAMBI, TGFB2, HAMP, BMP6*

## Data Availability

The raw sequence data are available from the Sequence Read Archive (SRA) database at the National Center for Biotechnology Information (NCBI) with the BioProject ID PRJNA882991.

## Supplementary Materials

The following supporting information can be downloaded at: https://www.mdpi.com/article/10.3390/ani13132208/s1. Figure S1: Principal component analysis of genotyped animals showing the diverse genetic background of the bulls; Table S1: An overview of RNA-Seq data for each sample on d0, d21, and d28; Figure S2: PCA, cluster dendrogram, and correlation matrix were performed for blood samples collected at d0 of the immunization protocol. Red points in the dot plot represent high-AMIR samples. Blue points in the dot plot represent low-AMIR samples; Figure S3: PCA, cluster dendrogram, and correlation matrix were performed for blood samples collected at d21 of the immunization protocol. Red points in the dot plot represent high-AMIR samples. Blue points in the dot plot represent low-AMIR samples; Figure S4: PCA, cluster dendrogram, and correlation matrix were performed for blood samples collected at d28 of the immunization protocol. Red points in the dot plot represent high-AMIR samples. Blue points in the dot plot represent low-AMIR samples. Supplementary File S2: Significantly enriched GO terms, Supplementary File S3: Significantly enriched KEGG pathways, Supplementary File S4: A summary of the genes used for Mfuzz analysis.
