# Supplementary material for: RNA-Seq Analysis of Peripheral Whole Blood from Dairy Bulls with High and Low Antibody-Mediated Immune Responses—A Preliminary Study
Source: Animals (Basel). 2023 Jul 5;13(13):2208. doi: 10.3390/ani13132208 (PMC10339907; doi:10.3390/ani13132208)
Supplement: Supplementary file 1 [file animals-13-02208-s001.zip › Supplementary_File_S1.pdf]

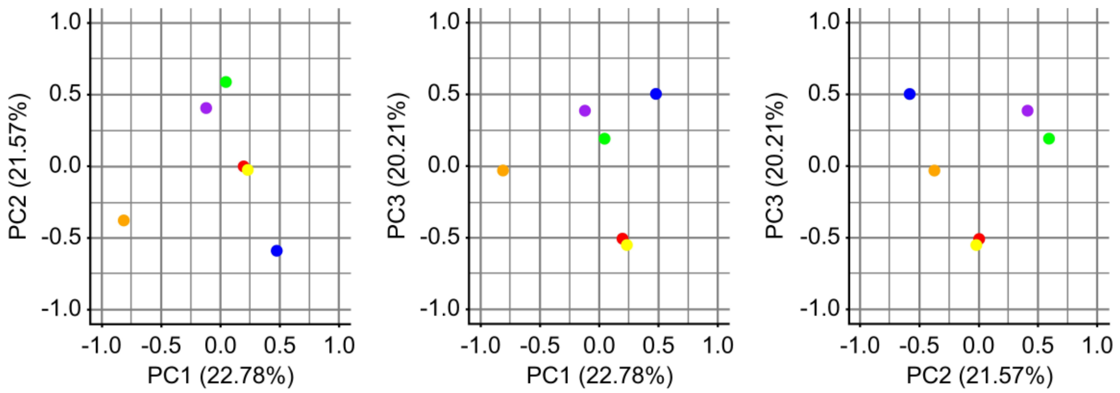

Figure S1. Principle component analysis of genotyped animals showing the diverse genetic background of the bulls.

Table S1. An overview of RNA-Seq data for each sample on d0, d21, and d28

Time point	Sample ID	Raw reads	Clean reads	Clean bases	Error rate (%)	Q30(%)	GC content (%)	Uniquely mapping rate (%)
d0	H1	43495220	42810380	6.42G	0.02	94.49	55.7	89.2
d0	H2	44360638	43579692	6.54G	0.02	94.55	52.67	90.28
d0	H3	44070966	43339170	6.5G	0.03	93.85	51.9	92.3
d0	L1	46258786	45533910	6.83G	0.02	94.46	52.09	90.73
d0	L2	46171190	45377994	6.81G	0.03	93.83	52.1	92.36
d21	H1	45900758	45203878	6.78G	0.03	93.83	52.37	93.35
d21	H2	47360010	46597954	6.99G	0.03	94.05	51.96	92.75
d21	H3	43368730	42185854	6.33G	0.02	95.06	52.79	90.35
d21	L1	46869544	46112462	6.92G	0.03	93.98	52.67	92.97
d21	L2	47352394	46416178	6.96G	0.03	93.82	52.32	92.18
d28	H1	45289710	44290126	6.64G	0.02	94.48	53.6	91.34
d28	H2	41285660	40397966	6.06G	0.02	94.91	52.74	90.96
d28	H3	43119940	41978048	6.3G	0.02	95.04	53.03	89.44
d28	L1	47200916	46347510	6.95G	0.02	94.7	53.67	91.13
d28	L2	46644960	45659144	6.85G	0.02	94.95	54	91.06

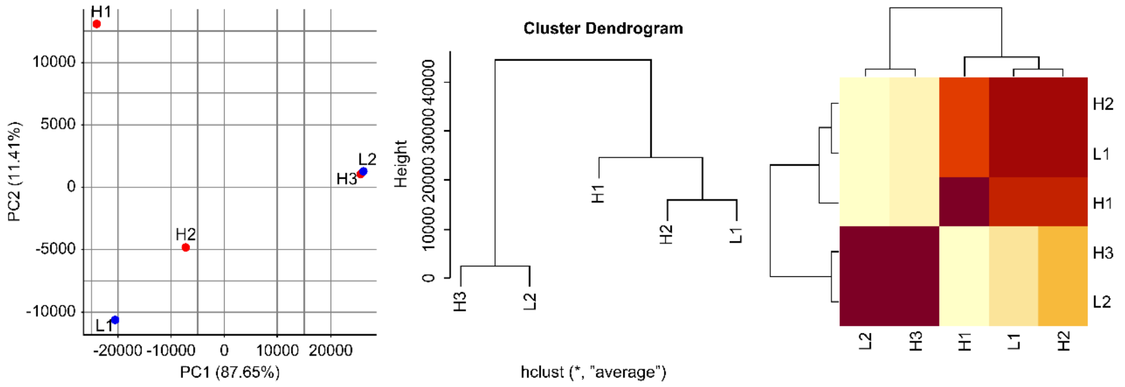

Figure S2. PCA, cluster dendrogram, and correlation matrix were performed for blood samples collected at d0 of the immunization protocol. Red points in the dot plot represent high-AMIR samples. Blue points in the dot plot represent low-AMIR samples.

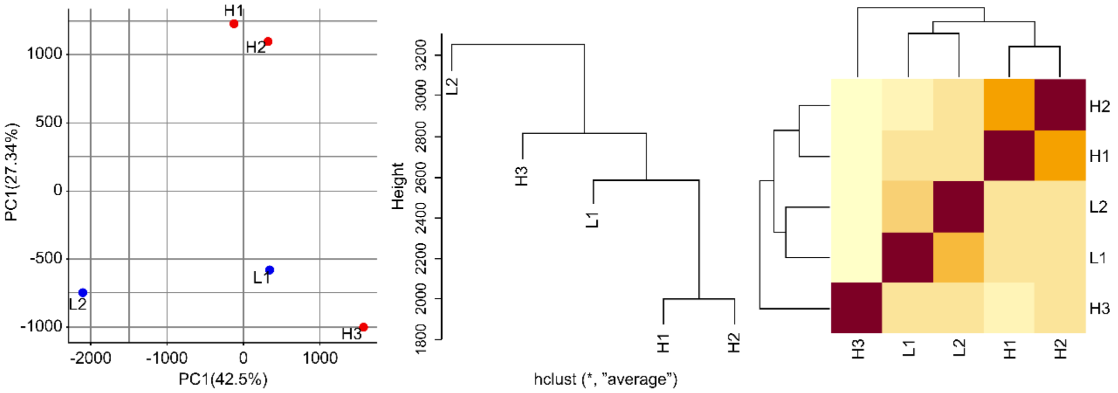

Figure S3. PCA, cluster dendrogram, and correlation matrix were performed for blood samples collected at d21 of the immunization protocol. Red points in the dot plot represent high-AMIR samples. Blue points in the dot plot represent low-AMIR samples.

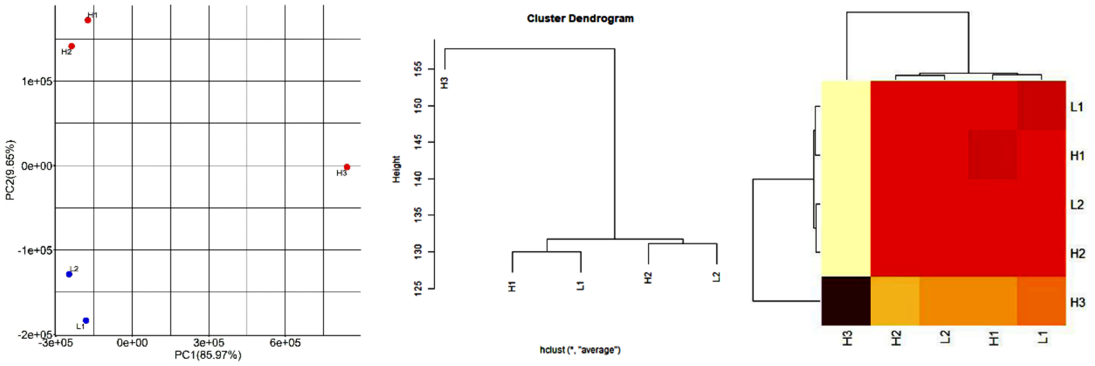

Figure S4. PCA, cluster dendrogram, and correlation matrix were performed for blood samples collected at d28 of the immunization protocol. Red points in the dot plot represent high-AMIR samples. Blue points in the dot plot represent low-AMIR samples.
